# Persistent poor glycaemic control in individuals with type 2 diabetes in developing countries: 12 years of real-world evidence of the International Diabetes Management Practices Study (IDMPS)

**DOI:** 10.1007/s00125-019-05078-3

**Published:** 2020-01-04

**Authors:** Pablo Aschner, Juan J. Gagliardino, Hasan Ilkova, Fernando Lavalle, Ambady Ramachandran, Jean Claude Mbanya, Marina Shestakova, Jean-Marc Chantelot, Juliana C. N. Chan

**Affiliations:** 1 Javeriana University School of Medicine, San Ignacio University Hospital, Carrera 7 # 40-62, Bogotá, 110231 Colombia; 2 CENEXA, Center of Experimental and Applied Endocrinology (La Plata National University National Scientific and Technical Research Council), La Plata, Argentina; 3 Division of Endocrinology Metabolism and Diabetes, Department of Internal Medicine, Cerrahpasa Medical Faculty, Istanbul University – Cerrahpasa, Istanbul, Turkey; 4 Facultad de Medicina, Universidad Autónoma de Nuevo León, Monterrey, Mexico; 5 India Diabetes Research Foundation, Dr. A. Ramachandran’s Diabetes Hospitals, Chennai, India; 6 Biotechnology Center, Doctoral School of Life Sciences, Health and Environment, University of Yaounde I, Yaounde, Cameroon; 7 Department of Medicine and Specialities, Faculty of Medicine and Biomedical Sciences, University of Yaounde I, Yaounde, Cameroon; 8 Endocrinology Research Center, Moscow, Russia; 9 Primary Care Medical China and Emerging Markets, Sanofi, Paris, France; 10 Department of Medicine and Therapeutics, Hong Kong Institute of Diabetes and Obesity and Li Ka Shing Institute of Health Science, The Chinese University of Hong Kong, The Prince of Wales Hospital, Shatin, Hong Kong SAR China

**Keywords:** Clinical diabetes, Education, Epidemiology, Healthcare delivery, Insulin therapy, Prediction and prevention of type 2 diabetes

## Abstract

**Aims/hypothesis:**

We evaluated the secular trend of glycaemic control in individuals with type 2 diabetes in developing countries, where data are limited.

**Methods:**

The International Diabetes Management Practices Study provides real-world evidence of patient profiles and diabetes care practices in developing countries in seven cross-sectional waves (2005–2017). At each wave, each physician collected data from ten consecutive participants with type 2 diabetes during a 2 week period. The primary objective of this analysis was to evaluate trends of glycaemic control over time.

**Results:**

A total of 66,088 individuals with type 2 diabetes were recruited by 6099 physicians from 49 countries. The proportion of participants with HbA_1c_ <53 mmol/mol (<7%) decreased from 36% in wave 1 (2005) to 30.1% in wave 7 (2017) (*p* < 0.0001). Compared with wave 1, the adjusted ORs of attaining HbA_1c_ ≤64 mmol/mol (≤8%) decreased significantly in waves 2, 5, 6 and 7 (*p* < 0.05). Over 80% of participants received oral glucose-lowering drugs, with declining use of sulfonylureas. Insulin use increased from 32.8% (wave 1) to 41.2% (wave 7) (*p* < 0.0001). The corresponding time to insulin initiation (mean ± SD) changed from 8.4 ± 6.9 in wave 1 to 8.3 ± 6.6 years in wave 7, while daily insulin dosage ranged from 0.39 ± 0.21 U/kg (wave 1) to 0.33 ± 0.19 U/kg (wave 7) for basal regimen and 0.70 ± 0.34 U/kg (wave 1) to 0.77 ± 0.33 (wave 7) U/kg for basal–bolus regimen. An increasing proportion of participants had ≥2 HbA_1c_ measurements within 12 months of enrolment (from 61.8% to 92.9%), and the proportion of participants receiving diabetes education (mainly delivered by physicians) also increased from 59.0% to 78.3%.

**Conclusions:**

In developing countries, glycaemic control in individuals with type 2 diabetes remained suboptimal over a 12 year period, indicating a need for system changes and better organisation of care to improve self-management and attainment of treatment goals.

**Electronic supplementary material:**

The online version of this article (10.1007/s00125-019-05078-3) contains peer-reviewed but unedited supplementary material, which is available to authorised users.

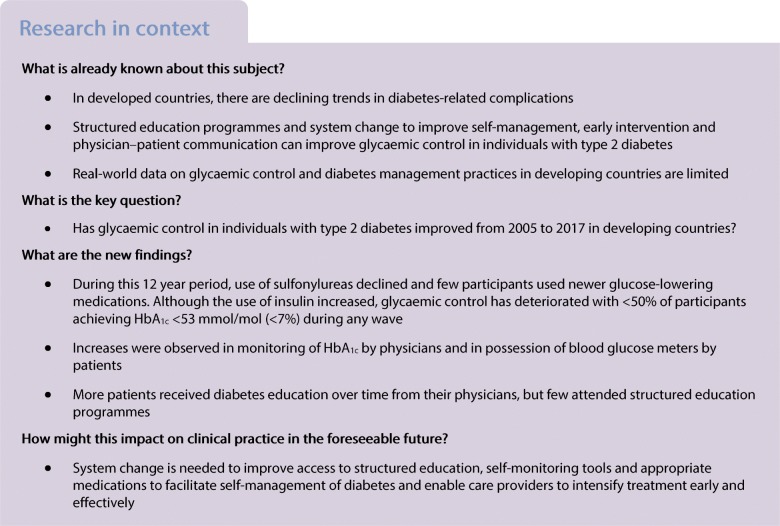



## Introduction

Diabetes affects approximately 463 million people worldwide, of whom 90% have type 2 diabetes, and the prevalence is expected to increase by 51% by 2045. A large number of people with diabetes live in developing regions, with estimates suggesting that 55 million people in the Middle East/North Africa, 32 million in South and Central America, 19 million in sub-Saharan Africa and 88 million in South East Asia have diabetes [1].

Poor glycaemic control can lead to an increased risk of blindness, end-stage renal disease, cardiovascular disease and lower limb amputations [2]. In 2012, 1.5 million deaths worldwide were directly caused by diabetes [2]. A further 2.2 million deaths were due to cardiovascular disease, chronic kidney disease and tuberculosis, associated with high blood glucose levels [2]. Optimal blood glucose control is therefore needed to reduce the risk of complications and premature death in developing regions, which have a high burden of diabetes and possess fewer resources to treat end-stage disease, creating a considerable impact on healthcare systems.

Over the last decade, there have been major advances in diabetes management, resulting in improved outcomes for individuals with type 2 diabetes. These include the introduction of novel technologies, newer oral glucose-lowering drugs (OGLD; e.g. sodium–glucose cotransporter [SGLT]_2_ inhibitors, dipeptidyl peptidase 4 inhibitors [DPP-4i] and glucagon-like peptide-1 receptor agonists [GLP-1 RA]), insulin therapies and delivery systems/devices.

While the majority of guidelines consider an HbA_1c_ goal of 53 mmol/mol (<7%) to be appropriate in most individuals with diabetes, use of individualised HbA_1c_ goals are recommended, based on patient preferences, characteristics, comorbidities and risk of adverse events [3–9]. These individualised goals can range from <48 mmol/mol (<6.5%) in individuals with low hypoglycaemia risk and <64 mmol/mol (<8%) in high-risk patients, such as those with a history of severe hypoglycaemia, extensive comorbidities or complications, older individuals or those with long-standing diabetes [3–9].

Self-management is a cornerstone in diabetes care and provision of diabetes education can improve self-management behaviours and glycaemic control [10–13]. Most professional bodies recommend the use of structured diabetes education and support programmes delivered by trained healthcare providers (HCPs), such as nurses, to improve self-management, and that these education programmes should be given at the time of diagnosis [3–9]. According to the International Diabetes Federation guidelines, for every primary care facility, at least one HCP should be trained as a diabetes educator, and these facilities should provide regular, structured group education to support individuals when needed [5].

In anticipation of increasing disease awareness, better care standards and technological advancements, the International Diabetes Management Practices Study (IDMPS) was designed to document and track patient profiles and patterns of care across time in developing countries, where data are limited. The IDMPS is the largest international observational study with the participation of over 6000 physicians from 49 countries across Africa, the Middle East, South Asia, Latin America, Asia and Eurasia. Data were collected using structured case report forms in a series of yearly ‘waves’, with each wave recruiting a different cohort of participants. The first wave of data collection began in 2005 and the most recent wave (wave 7) was completed in 2017. Apart from a standard dataset collected in all waves, each wave had a particular theme, such as understanding factors involved in glycaemic control (wave 1), healthcare resource utilisation (wave 2), barriers to insulin therapy (wave 3), hypoglycaemia (wave 4), symptoms of depression (wave 5), self-management (wave 6) and insulin discontinuation (wave 7) [10, 14–17].

Results from previous waves have demonstrated the low attainment of treatment goals for LDL-cholesterol (<2.6 mmol/l), BP (<130/80 mmHg) and HbA_1c_ (53 mmol/mol [<7%]) with only 25% of participants with type 1 diabetes and 36% of participants with type 2 diabetes achieving HbA_1c_ of 53 mmol/mol (<7%); <8% of participants achieved all three goals [14]. Data analysis from other waves has demonstrated the positive associations of good glycaemic control with patient education and self-monitoring of blood glucose (SMBG) [10], in addition to the positive association of diabetes-related complication rates and increased healthcare resource utilisation [15, 17].

The present analysis of real-world data describes glycaemic goal achievement, therapy use and care management practices in people with type 2 diabetes over time, from the first wave of IDMPS data collection (2005) to the most recent wave (2017).

## Methods

### Study design and participants

The IDMPS is an ongoing international, multicentre, non-interventional, observational study, which documents current practices in diabetes management. From 2005 to 2017, data were collected in seven individual waves, each wave enrolling different patients and physicians. At each wave, during a 2 week period, participating physicians were asked to enter details of the first five patients with type 1 diabetes (data not shown) and ten patients with type 2 diabetes who made a routine visit, using structured care report forms. Participating physicians included both general practitioners and specialists, providing they had experience of caring for individuals with diabetes and prescribing insulin therapy. Physicians were randomly selected following stratification for specialty. Study design and reporting format are in accordance with the recommended STROBE (Strengthening the Reporting of Observational Studies in Epidemiology) guidelines. Ethics approval was obtained from institutional review boards in each country. The study was conducted in accordance with the Declaration of Helsinki. Data from all seven waves are presented herein.

### Inclusion/exclusion criteria

Men and women above the lower legal age limit (country-specific) with a clinical diagnosis of type 2 diabetes who provided informed consent were eligible for inclusion. Exclusion criteria included concomitant participation in another clinical study, participation in a previous IDMPS wave and current receipt of temporary insulin therapy due to gestational diabetes, surgery or pancreatic cancer.

### Outcome measures

In this analysis, we examined the secular trend of glycaemic control and use of medications (i.e. insulin and/or OGLD) in individuals with type 2 diabetes over a 12 year period in 49 countries across Africa, the Middle East, South Asia, Latin America, Asia and Eurasia.

### Statistical analysis

#### Data collection

Patient information was recorded by the attending physician using individual case report forms during the 2 week study period.

#### Baseline variables and demographic characteristics

Evaluation variables were recorded by participating physicians and included the proportion of participants attaining recommended and individualised glycaemic goals according to their characteristics and treatments. Due to the non-interventional nature of the survey, no safety data were collected. Spontaneous adverse drug reactions were reported according to country-specific regulations.

#### Analysis populations

All individuals who fulfilled eligibility criteria and had no missing data concerning the treatment for diabetes defined as OGLD [yes/no] and insulin [yes/no] were included in the eligible population for analysis. Sample size was estimated assuming that insulin is the least prescribed therapy (in terms of proportion) to give an absolute precision of 20% and a CI of 95%. Data were analysed in the overall type 2 diabetes population and stratified by treatment subgroups including OGLD only, OGLD + insulin and insulin only.

#### Data analyses

Results presented are based on real-world data from all seven waves. Quantitative variables are expressed as mean ± SD and qualitative variables are expressed as numbers and percentages. No imputation was made for missing data, with the exception of age (missing date of birth was set to ‘15’ and missing month of birth was set to ‘June’); missing data were not counted in the percentages. Trend analyses were conducted on all-wave data to assess the significance of changes over time for key variables including last HbA_1c_ measurement, HbA_1c_ goal attainment, current insulin use, BMI and waist circumference recorded at enrolment. Statistical approaches for trends analyses varied depending on the data sets: categorical variables (e.g. HbA_1c_ <53/≥53 mmol/mol [<7/≥7%] or yes/no) were assessed using a two-sided Cochran–Armitage test to investigate the relationship between study waves and the variables of interest, assuming a no-trend null hypothesis; continuous variables were assessed using a least-squares fit value (R^2^). We used logistic regression models to explore the trend of attainment of HbA_1c_ <53 mmol/mol (<7%) over time across all waves; data were adjusted for age, sex, treatment regimen, disease duration and diabetes education. While an HbA_1c_ goal of <53 mmol/mol (<7%) is considered appropriate for many people with type 2 diabetes, guidelines state the importance of individualised goals, the highest of which is <64 mmol/mol (<8%) [3–9]. Consequently, achievement of HbA_1c_ <64 mmol/mol (<8%) was also assessed. Owing to the heterogeneity of study populations across regions and differences in regional participation across waves, this further analysis included region as an adjustment, in addition to the adjustments used for analysis of HbA_1c_ <53 mmol/mol (<7%), and analyses were performed comparing waves 2–7 individually using wave 1 as a reference. Statistical analyses were performed using SAS (software version 9.4 [2016], SAS Institute, Cary, NC, USA).

## Results

### Study population and baseline characteristics

#### Patient population

We included 66,088 individuals with type 2 diabetes from seven waves of data collection; of these, 42,171 (63.8%) received OGLD only, 14,529 (22.0%) received OGLD + insulin and 7566 (11.4%) received insulin only. Patients treated with meal plan/physical activity alone (*n* = 1685, 2.5%) and those who did not use OGLD, insulin or diet for their diabetes (*n* = 137, 0.2%) were excluded from this analysis. A full list of participating countries is listed in electronic supplementary material (ESM) Table 1. A total of 49 countries were included in the seven waves of data collection, grouped into regions with the composition of each region varying by wave depending on logistic availability.

#### Baseline demographics and characteristics

During the 12 year period, the mean age of participants with type 2 diabetes was ~58 years and ~52% were women. Disease duration increased from 8.3 years in wave 1 to 9.8 years in wave 7, accompanied by a rising trend of BMI in the whole group and in the treatment subgroups (data for therapy subgroups not shown). The proportion of participants with hypertension and dyslipidaemia increased significantly over time by 5.3% and 19.6%, respectively (*p* < 0.0001 for both) (Table 1).

**Table 1:** Clinical profiles, care processes and self-monitoring in participants with type 2 diabetes at enrolment between 2005 and 2017

Characteristic	Wave 1 (2005) *N* = 9918	Wave 2 (2006) *N* = 17,232	Wave 3 (2008) *N* = 12,210	Wave 4 (2010) *N* = 5343	Wave 5 (2011–12) *N* = 9603	Wave 6 (2013–14) *N* = 5479	Wave 7 (2016–17) *N* = 6303
Age, years (SD)	58.1 (11.5)	58.2 (11.8)	57.7 (11.8)	58.4 (11.9)	57.6 (11.2)	57.3 (10.7)	57.2 (11.1)
Female sex, n (%)	5130 (51.9)	8736 (52.2)	6458 (54.1)	2882 (54.3)	5117 (53.3)	3048 (55.6)	3291 (52.2)
Weight, kg (SD)	71.6 (14.9)	75.4 (16.0)	76.7 (16.2)	78.9 (16.5)	80.6 (16.2)	82.6 (16.7)	82.0 (16.7)
Disease duration, years	8.3 (7.1)	8.6 (7.8)	8.8 (7.7)	9.1 (8.1)	8.7 (7.3)	9.3 (7.1)	9.8 (7.4)
BMI, kg/m^2^ (SD)	27.1 (4.8)	28.5 (5.3)	29.0 (5.5)	29.8 (5.5)	29.6 (5.5)	30.2 (5.7)	29.8 (5.5)
Hypertension, n (%)	6029 (60.9)	10,681 (62.5)	7422 (61.0)	3254 (61.1)	6315 (66.0)	3623 (66.4)	4166 (66.2)
Dyslipidaemia, n (%)	4844 (49.3)	9609 (60.2)	7267 (62.5)	3485 (67.2)	5855 (63.4)	3449 (64.8)	3970 (68.9)
Last HbA_1c_ measurement, mmol/mol (SD)	61.5 (85.0)	63.2 (86.7)	62.3 (85.8)	62.6 (86.1)	64.4 (87.9)	64.1 (87.7)	64.6 (88.1)
Last HbA_1c_ measurement, % (SD)	7.8 (1.8)	7.9 (1.9)	7.9 (2.0)	7.9 (1.9)	8.0 (1.9)	8.0 (1.8)	8.1 (1.9)
HbA_1c_ testing, n (%)	6116 (61.8)	12,492 (76.5)	9217 (80.4)	4401 (85.5)	8399 (90.0)	4952 (92.1)	5719 (92.9)
Frequency of testing of HbA_1c_ during past year	1.6 (1.2)	2.2 (1.4)	2.3 (1.4)	2.2 (1.5)	2.2 (1.8)	2.2 (2.0)	2.2 (1.7)

Mean values are presented unless otherwise stated

Percentages were calculated for patients with available data; these varied by each category/wave

Hypertension and dyslipidaemia were defined (yes/no) according to the attending physician

#### Achievement of glycaemic goal

Mean HbA_1c_ varied by therapy type, with lower values in individuals on OGLD only compared with those treated with OGLD + insulin or insulin only (ESM Table 2). Last mean HbA_1c_ measurement increased slightly from 7.8% in wave 1 to 8.1% in wave 7 for the overall population, although this increase was not clinically meaningful. Similar slight increases were shown across the different therapy groups.

**Fig. 1:**
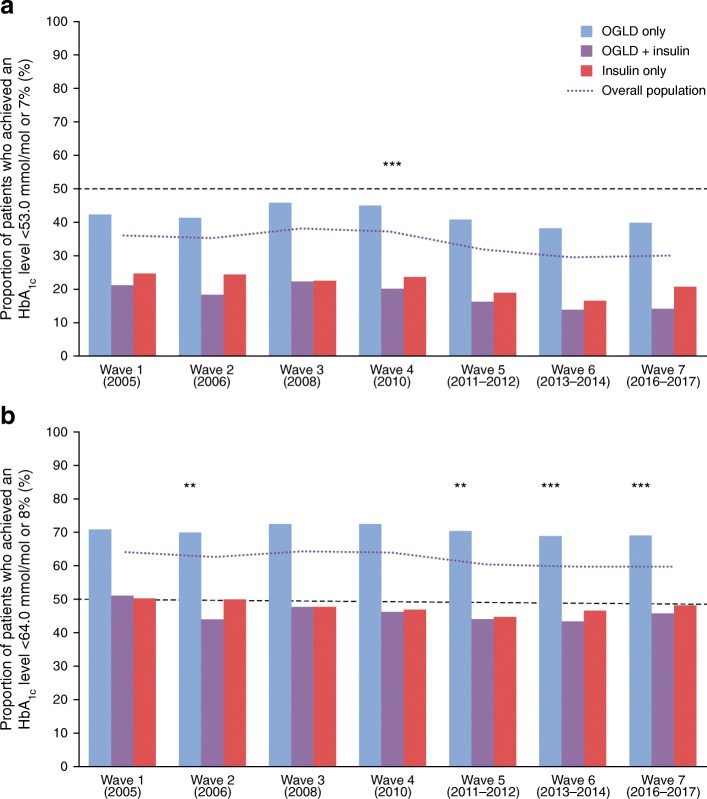
The proportion of participants attaining HbA_1c_ goal defined as: (**a**) <53 mmol/mol or <7%; and (**b**) <64 mmol/mol or <8%, between 2005 and 2017. The *p* values show test of significance for trend in HbA_1c_ goal achievement in the overall population: (**a**) over all waves; or (**b**) waves 2–7 vs reference wave 1. **p < 0.01, ***p < 0.001. A two-sided Cochran–Armitage test was used to investigate the relationship between study waves and the variables of interest, assuming a no-trend null hypothesis: (**a**) *p* < 0.0001 for trend over all waves; (**b**) *p* = 0.0036 for wave 2 vs wave 1, *p* = 0.2991 for wave 3 vs wave 1, *p* = 0.0514 for wave 4 vs wave 1, *p* = 0.0011 for wave 5 vs wave 1, *p* = 0.0006 for wave 6 vs wave 1 and *p* = 0.0017 for wave 7 vs wave 1. HbA_1c_ goal achievement data were missing for 3893 participants in wave 1, 5084 participants in wave 2, 3150 participants in wave 3, 961 participants in wave 4, 1256 participants in wave 5, 548 participants in wave 6 and 608 participants in wave 7

Overall, <50% of participants attained HbA_1c_ goal (<53 mmol/mol [<7%]) in any wave, irrespective of therapy subgroup (Fig. 1); no difference in glycaemic control was seen between patients treated by general practitioners and patients treated by specialists (data not shown). Among participants treated with insulin, <30% achieved HbA_1c_ <53 mmol/mol (<7%), regardless of whether they received insulin alone or in combination with OGLD (Fig. 1a). Trends analyses showed a significant decline in HbA_1c_ <53 mmol/mol (<7%) goal achievement in the overall population (*p* < 0.0001) and treatment subgroups (*p* < 0.0001 for all).

Using HbA_1c_ ≤64 mmol/mol (≤8%) as an alternative goal, the adjusted OR of attaining HbA_1c_ goal (using wave 1 as reference) was significantly lower in waves 2, 5, 6 and 7 (*p* < 0.05 for all; Fig. 1b). Female participants, those with disease duration >10 years and individuals living in East Europe/Eurasia were less likely to attain HbA_1c_ ≤ 64 mmol/mol (≤8%) (Table 2). Age was also a factor in achievement of this higher glycaemic goal, with individuals aged >40 years being significantly more likely to achieve it compared with participants aged ≤40 years; the likelihood of achieving HbA_1c_ ≤64 mmol/mol (≤8%) increased for each decade over the age of 40 years (Table 2). Proportions of participants achieving various HbA_1c_ goals in each wave are shown in ESM Table 3.

**Table 2:** Logistic regression analysis of glycaemic goal achievement defined as HbA_1c_ ≤64 mmol/mol (≤8%)

Comparison	*p* value	OR (95% CI)
Wave^a^		
Wave 2 vs wave 1	0.0036	0.90 (0.84, 0.97)
Wave 3 vs wave 1	0.2991	0.96 (0.90, 1.04)
Wave 4 vs wave 1	0.0514	0.92 (0.84, 1.00)
Wave 5 vs wave 1	0.0011	0.89 (0.83, 0.95)
Wave 6 vs wave 1	0.0006	0.87 (0.80, 0.94)
Wave 7 vs wave 1	0.0017	0.89 (0.82, 0.96)
Age (years)		
40–50 vs ≤40	0.0161	1.11 (1.02, 1.21)
50–60 vs ≤40	<0.0001	1.22 (1.13, 1.33)
60–70 vs ≤40	<0.0001	1.59 (1.46, 1.72)
>70 vs ≤40	<0.0001	2.08 (1.89, 2.28)
Men vs women	<0.0001	1.10 (1.06, 1.14)
Time since diagnosis ≤10 vs >10 years	<0.0001	1.80 (1.73, 1.88)
Region		
Africa/Asia vs Eurasia/East Europe	<0.0001	1.26 (1.19, 1.33)
Middle East/Latin America vs Eurasia/East Europe	<0.0001	1.39 (1.32, 1.47)

^a^HbA_1c_ goal achievement data were missing for 3893 participants in wave 1, 5084 participants in wave 2, 3150 participants in wave 3, 961 participants in wave 4, 1256 participants in wave 5, 548 participants in wave 6 and 608 participants in wave 7

#### OGLD therapy

Overall, >80% of participants were treated with OGLD with or without insulin (Table 3). The use of sulfonylurea monotherapy decreased over time, while use of metformin monotherapy increased. Overall, use of other types of OGLD was low (14.5–20.0% of participants; Table 3). Metformin was the most commonly used monotherapy, while sulfonylureas and DPP-4is were often second- or third-line OGLD (ESM Table 4).

**Table 3:** Distribution of use of OGLD in participants with type 2 diabetes between 2005 and 2017

Characteristic	Wave 1 (2005) *N* = 9918	Wave 2 (2006) *N* = 17,232	Wave 3 (2008) *N* = 12,210	Wave 4 (2010) *N* = 5343	Wave 5 (2011–12) *N* = 9603	Wave 6 (2013–14) *N* = 5479	Wave 7 (2016–17) *N* = 6303
Proportion of participants treated with OGLD, %	81.0	84.9	87.1	91.8	85.6	87.0	88.4
Number of OGLD treatments received							
1 OGLD therapy, *n* (%)	3527 (35.7)	6353 (36.9)	4293 (36.0)	2304 (43.6)	4542 (47.3)	1876 (34.2)	2125 (34.6)
>1 OGLD therapy, *n* (%)	4478 (45.3)	8248 (47.9)	6062 (50.8)	2536 (48.0)	3672 (38.2)	2890 (52.8)	3295 (53.6)
Type of OGLD treatment received^a^							
Metformin alone, *n* (%)	–	3258 (18.9)	2517 (21.1)	1246 (23.6)	2342 (24.4)	1459 (26.6)	1638 (26.7)
Sulfonylureas alone, *n* (%)	–	2371 (13.8)	1331 (11.2)	307 (5.8)	714 (7.4)	306 (5.6)	257 (4.2)
Metformin + sulfonylureas, *n* (%)	–	6478 (37.6)	4726 (39.6)	2195 (41.6)	3749 (39.0)	2199 (40.1)	2281 (37.2)
Other, *n* (%)^b^	–	2494 (14.5)	1781 (14.9)	1092 (20.7)	1409 (14.7)	802 (14.6)	1223 (20.0)

Percentages were calculated for patients with available data; these varied by each category/wave

^a^Data not available for wave 1

^b^Detailed information on ‘Other’ therapies is available in ESM Table 4

#### Insulin therapy

**Fig. 2:**
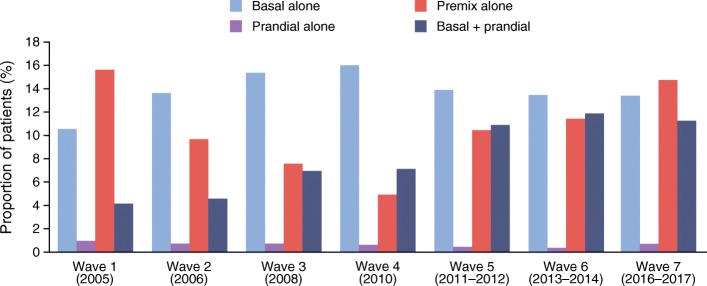
Changes in use of insulin regimens in type 2 diabetes between 2005 and 2017

The proportion of participants treated with insulin increased from 32.8% in wave 1 to 41.2% in wave 7 (*p* < 0.0001; Table 4). Most participants received either basal insulin alone or premix insulin alone in wave 1 with a similar pattern observed in wave 7. The use of premix insulin declined from wave 1 to 4 and increased again from waves 4 to 7, while the use of basal + prandial insulin has increased almost threefold over time (Fig. 2/ESM Fig. 1). Analysis of insulin type (human vs analogue; waves 6 and 7 only; ESM Table 5) revealed that use of long-acting basal insulin analogues increased between waves 6 and 7, but human intermediate-acting insulin was still used by 24.4% of participants receiving basal insulin in wave 7. Approximately 50% of participants using prandial insulin used short-acting analogues in waves 6 and 7 and 61.0% of those using premix insulin received human insulin, although this proportion decreased to 57.8% by wave 7.

**Table 4:** Distribution of insulin regimens and insulin dose in participants with type 2 diabetes between 2005 and 2017

Characteristic	Wave 1 (2005) *N* = 9918	Wave 2 (2006) *N* = 17,232	Wave 3 (2008) *N* = 12,210	Wave 4 (2010) *N* = 5343	Wave 5 (2011–12) *N* = 9603	Wave 6 (2013–14) *N* = 5479	Wave 7 (2016–17) *N* = 6303
Proportion of participants treated with insulin, %	32.8	29.8	31.5	31.8	36.7	37.9	41.2
Time to initiation of insulin treatment, years	8.4 (6.9)	9.3 (7.5)	9.6 (7.6)	10.0 (7.8)	8.4 (6.8)	8.4 (6.4)	8.3 (6.6)
Time on insulin treatment, years	5.8 (5.1)	5.0 (4.7)	3.5 (4.4)	3.5 (4.3)	3.8 (4.4)	4.5 (4.7)	4.7 (4.8)
Daily insulin dose, U							
Basal alone	26.6 (14.9)	28.6 (16.9)	28.2 (16.1)	26.3 (13.7)	25.5 (13.4)	26.7 (13.8)	26.4 (15.6)
Prandial alone	35.6 (19.4)	36.1 (23.8)	30.0 (17.4)	33.6 (21.8)	28.8 (17.7)	24.2 (16.0)	38.9 (35.8)
Premix alone	36.6 (17.0)	42.1 (20.2)	44.1 (20.8)	48.8 (21.8)	42.4 (19.9)	44.6 (22.4)	44.9 (24.7)
Basal + prandial	49.9 (23.1)	56.6 (27.4)	56.2 (27.8)	57.2 (27.3)	56.9 (28.6)	62.2 (25.8)	64.0 (28.9)
Daily insulin dose (weight-adjusted), U/kg							
Basal alone	0.39 (0.21)	0.39 (0.23)	0.38 (0.21)	0.36 (0.19)	0.32 (0.16)	0.33 (0.16)	0.33 (0.19)
Prandial alone	0.54 (0.29)	0.50 (0.31)	0.40 (0.22)	0.40 (0.22)	0.40 (0.27)	0.31 (0.25)	0.47 (0.32)
Premix alone	0.53 (0.24)	0.56 (0.25)	0.59 (0.27)	0.62 (0.26)	0.54 (0.25)	0.55 (0.24)	0.56 (0.28)
Basal + prandial	0.70 (0.34)	0.73 (0.34)	0.73 (0.33)	0.72 (0.34)	0.69 (0.32)	0.74 (0.30)	0.77 (0.33)

Values are presented as mean (SD) unless otherwise stated. Percentages were calculated for patients with available data; these varied by each category/wave

The mean time to insulin initiation in the overall type 2 diabetes population was >8 years across all waves and remained stable over time (Table 4). Similar results were seen when assessing the insulin only and insulin + OGLD therapy subgroups (data not shown). The time on insulin treatment prior to study inclusion declined over time in all individuals treated with insulin (Table 4), especially in the OGLD + insulin subgroup (data not shown). The mean daily dose of insulin increased in all insulin regimens except for basal. The mean daily dose for the overall insulin-treated population, adjusted for body weight (U/kg), increased for premix insulin alone and basal + prandial but decreased for basal alone and prandial alone (Table 4).

#### Blood glucose monitoring and diabetes education

**Fig. 3:**
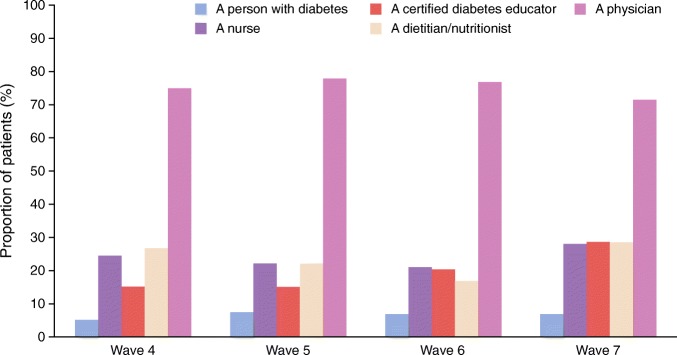
Distribution of sources of education in participants who received diabetes-related education. Data on the provider of diabetes education were not captured in waves 1–3

The proportion of individuals who had HbA_1c_ testing increased from wave 1 (61.8%) to wave 7 (92.9%), with screening typically occurring twice a year (Table 1/ESM Table 2). Participants treated with insulin were increasingly likely over time to own a glucose meter, but this was not reflected in the performance of SMBG; cost was increasingly cited as a limiting factor for regular SMBG testing (ESM Table 6). The overall proportion of participants receiving diabetes education increased over time, irrespective of therapy subgroup (ESM Table 7). However, based on data from waves 4–7, education was mainly provided on an individual basis by physicians with very few individuals attending structured diabetes education courses (Fig. 3).

## Discussion

Glycaemic control in developing countries has been persistently poor over the past 12 years and is growing steadily worse, based on real-world data captured in this large international observational study involving over 66,000 individuals with type 2 diabetes. This situation has developed despite multiple advances in the field of diabetes management, including the development of new medications proven to improve diabetes control and clinical outcomes [18].

Most participants in this study were aged 40–65 with 8–9 years of diabetes duration, with over 80% of participants receiving OGLD. While the use of sulfonylurea monotherapy declined over time, use of metformin monotherapy increased over time and metformin was the most commonly used monotherapy, in line with guideline recommendations [3–9]. Few participants received newer agents, such as SGLT_2_ inhibitors, although it should be noted that these therapies were approved relatively recently in many developing countries; as such, data on their use are only available for wave 7. The proportion of individuals receiving insulin also increased over time, with use of basal + prandial insulin showing a marked increase; despite this rise, however, there was no improvement in glycaemic control. Newer insulin analogue therapies can provide clinical benefits in terms of reduced glycaemic variability or hypoglycaemia risk [19], which may facilitate individuals to achieve glycaemic control; however, in developing countries, these analogues may not be readily available due to issues of access or cost. Data from wave 7 highlighted that a substantial proportion of participants still received human intermediate-acting insulins (24.4%), although long-acting analogue use increased between waves 6 and 7. Participants receiving prandial insulin were mainly using human regular insulins or premix insulins.

Overall, <50% of participants achieved a glycaemic goal of HbA_1c_ <53 mmol/mol (<7%), and <70% achieved HbA_1c_ <64 mmol/mol (8%). Furthermore, only 15–25% or 45–50% of participants treated with insulin achieved either the HbA_1c_ <53 mmol/mol (<7%) goal or the HbA_1c_ <64 mmol/mol (8%) goals, respectively. These results might be ascribed to a combination of delayed and inappropriate insulin regimen prescription. Logistic regression analysis was used to confirm the declining trend of HbA_1c_ <64 mmol/mol (<8%) goal attainment over time. The odds of goal attainment compared with wave 1 were significantly lower for waves 2, 5, 6 and 7 (*p* < 0.05 for all). Other patient groups (including younger individuals [≤40 years], women, those with longer disease duration [>10 years] and those from Europe/Eurasia) were also less likely to achieve the HbA_1c_ goal compared with their counterparts. Visits to general practitioners or specialists did not seem to influence achievement of glycaemic goal; any potential difference may have been attenuated by study selection criteria requiring all physicians to have prior experience in prescribing insulin. Additionally, individuals with poor glycaemic control or advanced disease progression might tend to seek specialist care.

Although there was an increase in the proportion of individuals receiving diabetes education from physicians, very few received structured diabetes education courses delivered by nurses, dietitians or certified diabetes instructors. This may potentially result in a lack of sufficient contact time to help individuals deal with day-to-day concerns.

These original findings from developing countries concur with other reports based on IDMPS data [14, 20], and are in line with data from developed countries indicating poor rates of glycaemic goal attainment (<53 mmol/mol [<7%]; ~20–40%) [21, 22], indicating that the challenge of attaining good glycaemic control is universal. It is concerning that glycaemic control remains poor, given the increased use of insulin over time; it should be noted that the insulin dose of 0.3–0.7 U/kg was comparable if not higher than that used in clinical trial settings [23–25]. In this survey, the mean time to insulin initiation was 8 years, similar to that reported in developed countries [26, 27].

Considering that >50% of participants in this study receiving OGLD and/or insulin treatment displayed an HbA_1c_ value >53 mmol/mol (>7%), this is a clear indicator that the initiation and intensification of insulin remain a major barrier in real-world practice. The discrepancy between doses prescribed and glycaemic control suggests possible patient non-adherence, although this was not formally tested. In this regard, quality improvement programmes implemented at a system level have been shown to improve control of cardiometabolic risk factors and clinical outcomes in community settings; such programmes include multidisciplinary care (with training programmes for physicians and nurses), risk-stratified care planning and regular structured/scheduled assessment of metabolic control and vascular complications [28–31]. The provision of professional diabetes education for HCPs starting in medical colleges, together with postgraduate training, is critically important to build capacity to help HCPs in educating patients and improving care. An alternative (ideally complementary) approach would be to make changes to the practice environment to provide more integrated multidisciplinary care, to ultimately provide sustained improvements in care outcomes. Such an approach would be conducive to promoting trustworthy relationships and communication between patients and HCPs, and should encompass empathetic listening, tuition of self-management skills and ongoing support [32].

According to professional guidelines, individuals with diabetes should receive structured diabetes education and support programmes [3, 5]; such programmes have been shown to improve glycaemic control, as reported in previous waves of the IDMPS [10]. In this study, over 70% of physicians reported having offered education to their patients; however, physicians are often time-poor in comparison with other HCPs (e.g. nurses, dietitians or certified diabetes instructors), and time is a major factor for a successful diabetes education programme. A meta-analysis of the efficacy of self-management education on glycaemic control in individuals with type 2 diabetes reported that approximately 24 h of contact time (e.g. face to face visits, phone calls) with HCPs are needed to sustain a 1% reduction in HbA_1c_ over a 12 month period [33]. There are also grounds to argue that regular follow-ups are needed, as results from a recent meta-review indicate that post-intervention improvements in HbA_1c_ persist until 6 months but tend to attenuate after 12 or 24 months [34]. Consultation times may also be inadequate, perhaps partly due to the pressures of the increasing global population of people with diabetes which may increase the number of patients each physician treats. A recent meta-analysis of primary care physician consultation time (spanning developed and developing countries) reported that consultation time was less than 5 min in 18 of the included countries, accounting for approximately 50% of the global population, with consultation length proportional to per capita health spending [35]. Such data as these indicate a strong need for additional support to engage patients and emphasise the importance of time spent talking to individuals about their needs and concerns. However, in our survey, <20% of participants received a structured educational diabetes programme from any source, which could be a contributing factor to the poor glycaemic goal achievement observed herein. We suggest that promising methods to improve glycaemic control would include training of nurses and dietitians in the provision of diabetes education, a general increase in the number of certified diabetes instructors and the delivery of such education through structured programmes.

The affordability and ownership of blood glucose monitoring accessories (e.g. blood glucose monitors and test strips) are important factors for improving self-management. In this study, an increasing proportion of participants possessed SMBG monitors over time, but there was also a rise in the number of individuals citing cost as a limiting factor for regular SMBG; in those treated with insulin, where a greater frequency of SMBG is needed, the high cost of strips may be a deterrent. Considered together, clinical inertia, insufficient access to structured diabetes education, infrequent SMBG and high cost of monitoring accessories/medications may all contribute to persistently poor glycaemic control in individuals with type 2 diabetes. A combination of factors ascribed to patients (e.g. fear of hypoglycaemia/injections, complex treatment regimen, polypharmacy), physicians (e.g. poor/ineffective communication, insufficient knowledge and support) or healthcare systems (e.g. lack of time/resource for physicians, lack of tools for patient/physician to monitor insulin titration, lack of medical coverage), may be particularly relevant in developing countries where development of infrastructure and capacity cannot cope with the rapid rate of increase in diabetes [36, 37]. Other patient-related factors, such as older age, higher education level and short disease duration, have also been reported to be associated with improved glycaemic control [38, 39]; it is possible that lifestyle factors, acceptance of diagnosis and adherence may all contribute in these cases.

The present study has some limitations. The cross-sectional nature of the survey provides a ‘snapshot’ of practice at any one time; therefore, these observations only allow us to form hypotheses, and cannot infer causality. The self-selecting nature of the patient population should be considered, as those with the poorest control are most likely to visit the clinic; therefore, glycaemic control in the general population may be higher than that shown here. All data were completed by the attending physicians with potential bias in interpretation and recall. Due to the pragmatic nature of the survey, no adjudication was conducted for the reported complications. The varying degrees of local support in implementing the survey also mean that different countries/regions were included/excluded in different waves. These variables add to the heterogeneity of practice, although we have adjusted for major variables (notably age, sex, disease duration and region) when analysing secular trends. Despite these limitations, we believe that aspects of this study such as its large, global population, long duration (12 years) and the practice of structured data collection using predefined variables have provided valuable real-world evidence from areas hit hardest by the diabetes epidemic, where data are lacking to inform practice and policies.

In conclusion, in this 12 year study, poor and worsening glycaemic control was observed in individuals with type 2 diabetes. These real-world data highlight an urgent need for improvement in practice environments, workflow and team structure; such amendments would allow early assessment of patients and identification of unmet needs, thus empowering individuals to improve self-management and consequently glycaemic control. These changes will need to be supplemented by institutional support through capacity building, and policies that promote good diabetes care; these would ideally encompass improved accessibility to and affordability of medications and monitoring accessories, in both developing countries and subpopulations of patients with poor literacy/low incomes in developed countries [5, 40]. Altogether, these could lead to a successful improvement in diabetes care and clinical outcomes in individuals with type 2 diabetes living in developing countries.

## Electronic supplementary material

ESM (PDF 277 kb)

## Data Availability

Qualified researchers may request access to related study documents, which will be redacted to protect the privacy of study participants. Further details on Sanofi’s data sharing criteria, eligible studies, and process for requesting access can be found at: https://www.clinicalstudydatarequest.com.

## Abbreviations

DPP-4i: Dipeptidyl peptidase 4 inhibitor
HCP: Healthcare provider
IDMPS: International Diabetes Management Practices Study
OGLD: Oral glucose-lowering drugs
SGLT: Sodium–glucose cotransporter
SMBG: Self-monitoring of blood glucose
