# Supplementary material for: Persistent poor glycaemic control in individuals with type 2 diabetes in developing countries: 12 years of real-world evidence of the International Diabetes Management Practices Study (IDMPS)
Source: Diabetologia. 2020 Jan 4;63(4):711–21. doi: 10.1007/s00125-019-05078-3 (PMC7054372; doi:10.1007/s00125-019-05078-3)
Supplement: Supplementary file 1 — (PDF 277 kb) [file 125_2019_5078_MOESM1_ESM.pdf]

Electronic Supplementary Material (ESM)

ESM Table 1. Included countries, by region

Region	*Waves of participation*	*Included countries*[a]
Africa	All	Algeria, Morocco, Tunisia, Egypt, South Africa, Senegal, Ivory Coast, Cameroon, Nigeria, Kenya, Zimbabwe, Madagascar, Democratic Republic of Congo
Asia	1–3, 5–7	China, Hong Kong, Indonesia, Thailand, Malaysia, Taiwan, Korea, India, Pakistan, Bangladesh
Eurasia	5–7	Russia, Ukraine, Kazakhstan, Uzbekistan, Azerbaijan, Georgia
Europe	1–5	Bosnia and Herzegovina, Romania, Bulgaria, Turkey
Latin America	1–5	Argentina, Colombia, Venezuela, Ecuador, Chile, Guatemala, Panama, Dominican Republic, Mexico
Middle East	2–7	United Arab Emirates, Kuwait, Iran, Iraq, Kingdom of Saudi Arabia, Jordan, Lebanon

[a]Not all countries in each region were included in each wave

ESM Table 2. HbA_{1c} screening, frequency and measurements by therapy type subgroup

Tests for HbA_{1c}	*Wave 1 (2005) N=9918*	*Wave 2 (2006) N=17,232*	*Wave 3 (2008) N=12,210*	*Wave 4 (2010) N=5343*	*Wave 5 (2011–12) N=9603*	*Wave 6 (2013–14) N=5479*	*Wave 7 (2016–17) N=6303*
OGLDs only	N=6389	N=11,403	N=7979	N=3530	N=5912	N=3321	N=3637
Patients screened, n (%)	3767 (59.1)	7998 (74.2)	5910 (79.0)	2869 (84.3)	5105 (88.6)	3007 (92.0)	3284 (92.6)
Frequency of testing during past year	1.6 (1.2)	2.2 (1.4)	2.2 (1.4)	2.1 (1.6)	2.1 (2.1)	2.2 (1.6)	2.2 (1.9)
Last measurement (%)	7.50 (1.66)	7.62 (1.79)	7.50 (1.78)	7.53 (1.67)	7.65 (1.77)	7.67 (1.68)	7.65 (1.72)
OGLDs + insulin	N=1632	N=3220	N=2617	N=1374	N=2304	N=1446	N=1936
Patients screened, n (%)	1147 (70.5)	2672 (86.6)	2159 (86.7)	1192 (89.7)	2127 (94.1)	1348 (94.7)	1793 (94.2)
Frequency of testing during past year	1.7 (1.2)	2.3 (1.4)	2.5 (1.4)	2.4 (1.4)	2.3 (1.2)	2.3 (2.1)	2.3 (1.4)
Last measurement (%)	8.29 (1.77)	8.65 (1.98)	8.56 (2.08)	8.60 (1.93)	8.63 (1.92)	8.63 (1.75)	8.62 (1.84)
Insulin only	N=1600	N=1911	N=1222	N=323	N=1217	N=633	N=660
Patients screened, n (%)	1032 (64.9)	1410 (78.6)	931 (82.8)	263 (85.9)	1043 (90.5)	534 (87.8)	581 (90.8)
Frequency of testing during past year	1.5 (1.2)	2.0 (1.5)	2.5 (1.6)	2.3 (1.2)	2.1 (1.3)	2.2 (3.4)	2.0 (1.2)
Last measurement (%)	8.38 (1.96)	8.50 (2.13)	8.53 (2.03)	8.62 (2.21)	8.70 (2.08)	8.50 (1.82)	8.75 (2.28)

Mean (SD) values are presented unless otherwise stated. Percentages were calculated for patients with available data; these varied by each category/wave. OGLD, oral glucose-lowering drug; SD, standard deviation

ESM Table 3. Proportion of patients achieving different HbA_{1c} goals in each wave

HbA_{1c}	*Wave 1 (2005) N=9918*	*Wave 2 (2006) N=17,232*	*Wave 3 (2008) N=12,210*	*Wave 4 (2010) N=5343*	*Wave 5 (2011–12) N=9603*	*Wave 6 (2013–14) N=5479*	*Wave 7 (2016–17) N=6303*
<53 mmol/mol (<7 %)	2168 (36.0)	4275 (35.2)	3446 (38.0)	1625 (37.1)	2658 (31.8)	1449 (29.4)	1716 (30.1)
53–64 mmol/mol (7–8 %)	1678 (27.9)	3304 (27.2)	2358 (26.0)	1166 (26.6)	2366 (28.3)	1480 (30.0)	1677 (29.4)
65–75 mmol/mol (8–9 %)	971 (16.1)	1817 (15.0)	1292 (14.3)	648 (14.8)	1376 (16.5)	863 (17.5)	936 (16.4)
>75 mmol/mol (>9 %)	1208 (20.0)	2752 (22.7)	1964 (21.7)	943 (21.5)	1947 (23.3)	1139 (23.1)	1366 (24.0)

n (%) data are presented unless otherwise stated. Percentages were calculated for patients with available data; these varied by each category/wave.

ESM Table 4. Use of OGLDs split according to number of OGLD agents in patients with type 2 diabetes between wave 4 and wave 7 (2010–2017)

		Biguanides	*Sulphonylureas*	*DPP-4i*	*TZD*	*Alpha-glucosidase inhibitors*	*Glinides*	*SGLT-2i*[a]
Wave 4	1 OGLD	1246 (54.1)	307 (13.3)	54 (2.3)	54 (2.3)	15 (0.7)	18 (0.8)	–
	2 OGLDs	1682 (84.5)	1427 (71.7)	236 (11.9)	270 (13.6)	55 (2.8)	78 (3.9)	–
	>2 OGLDs	459 (84.1)	451 (82.6)	244 (44.7)	326 (59.7)	73 (13.4)	54 (9.9)	–
Wave 5	1 OGLD	2342 (51.6)	714 (15.7)	50 (1.1)	17 (0.4)	38 (0.8)	41 (0.9)	–
	2 OGLDs	2450 (81.6)	2335 (77.8)	217 (7.2)	200 (6.7)	136 (4.5)	106 (3.5)	–
	>2 OGLDs	571 (85.2)	532 (79.4)	271 (40.4)	299 (44.6)	172 (25.7)	47 (7.0)	–
Wave 6	1 OGLD	1459 (77.8)	306 (16.3)	87 (4.6)	7 (0.4)	3 (0.2)	14 (0.7)	–
	2 OGLDs	2200 (94.7)	1774 (76.4)	510 (22.0)	53 (2.3)	43 (1.9)	64 (2.8)	–
	>2 OGLDs	565 (99.5)	540 (95.1)	447 (78.7)	103 (18.1)	63 (11.1)	19 (3.3)	–
Wave 7	1 OGLD	1638 (77.1)	257 (12.1)	136 (6.4)	15 (0.7)	17 (0.8)	41 (1.9)	21 (1.0)
	2 OGLDs	2352 (94.5)	1708 (68.6)	697 (28.0)	35 (1.4)	41 (1.6)	82 (3.3)	63 (2.5)
	>2 OGLDs	793 (98.4)	703 (87.2)	605 (75.1)	96 (11.9)	104 (12.9)	28 (3.5)	89 (11.0)

n (%) data are presented unless otherwise stated. Percentages were calculated for patients with available data; these varied by each category/wave.

[a]Data on SGLT-2i use were only recorded in wave 7.

ESM Table 5. Types of insulin used (overall population)

	Wave 6 (2013–14) *N=5479*	*Wave 7 (2016–17)* *N=6303*
Basal insulin	N=1401	N=1576
Long-acting insulin analogue	930 (66.6)	1167 (74.1)
Intermediate human insulin	452 (32.4)	384 (24.4)
Biosimilar insulin	14 (1.0)	24 (1.5)
Prandial insulin	N=704	N=804
Short-acting insulin analogue	368 (52.3)	434 (54.0)
Rapid-acting human insulin	330 (46.9)	369 (46.0)
Biosimilar insulin	5 (0.7)	2 (0.2)
Premix insulin	N=674	N=993
Insulin analogue	262 (39.0)	411 (42.3)
Human insulin	409 (61.0)	561 (57.8)

n (%) data are presented unless otherwise stated. Percentages were calculated for patients with available data; these varied by each category/wave.

ESM Table 6. Blood glucose monitoring, for overall population (A) and by therapy type subgroup (B)

A

Characteristic	*Wave 1 (2005) N=9918*	*Wave 2 (2006) N=17,232*	*Wave 3 (2008) N=12,210*	*Wave 4 (2010) N=5343*	*Wave 5 (2011–12) N=9603*	*Wave 6 (2013–14) N=5479*	*Wave 7 (2016–17) N=6303*
Fasting blood glucose testing performed, n (%)	9458 (95.4)	15,158 (88.0)	11,694 (95.8)	5126 (95.9)	8654 (90.5)	4997(91.2)	5504 (87.3)
Possession of glucose meter, n (%)[†]	3274 (33.1)	\	5740 (47.4)	3166 (59.7)	5757 (60.4)	3671 (67.6)	4393 (70.3)
Cost as limiting factor for regular SMBG, n (%)[††]	\	\	2444 (43.8)	1431 (45.9)	2584 (47.0)	1869 (53.7)	2316 (55.3)

n (%) data are presented unless otherwise stated. Percentages were calculated for patients with available data; these varied by each category/wave.

SD, standard deviation; SMBG, self-monitoring blood glucose

[†]Data not available for Wave 2; [††]Data not available for Waves 1 and 2

B

Characteristic	*Wave 1 (2005) N=9918*	*Wave 2 (2006) N=17,232*	*Wave 3 (2008) N=12,210*	*Wave 4 (2010) N=5343*	*Wave 5 (2011–12) N=9603*	*Wave 6 (2013–14) N=5479*	*Wave 7 (2016–17) N=6303*
OGLDs only	N=6389	N=11,403	N=7979	N=3530	N=5912	N=3321	N=3637
Fasting blood glucose testing performed, n (%)	6110 (95.6)	10092 (88.5)	7663 (96.0)	3372 (95.5)	5292 (89.9)	3004 (90.5)	3151 (86.6)
Possession of glucose meter, n (%)†	1543 (24.2)	\	3089 (39.1)	1862 (53.2)	3051 (52.1)	2003 (61.0)	2267 (62.9)
Cost as limiting factor for regular SMBG, n (%)††	\	\	1239 (41.4)	825 (45.2)	1315 (45.0)	933 (49.2)	1150 (53.4)
OGLDs + insulin	N=1632	N=3220	N=2617	N=1374	N=2304	N=1446	N=1936
Fasting blood glucose testing performed, n (%)	1547 (94.8)	2753 (85.5)	2515 (96.1)	1331 (96.9)	2116 (92.3)	1341 (92.7)	1716 (88.6)
Possession of glucose meter, n (%)†	859 (52.7)	\	1758 (67.6)	1019 (74.3)	1747 (76.3)	1151 (80.3)	1587 (82.5)
Cost as limiting factor for regular SMBG, n (%)††	\	\	788 (45.8)	463 (45.8)	810 (48.9)	619 (57.0)	845 (55.7)
Insulin only	N=1600	N=1911	N=1222	N=323	N=1217	N=633	N=660
Fasting blood glucose testing performed, n (%)	1525 (95.3)	1718 (89.9)	1151 (94.2)	314 (97.2)	1106 (91.3)	588 (92.9)	580 (87.9)
Possession of glucose meter, n (%)†	805 (50.4)	\	806 (66.3)	252 (78.3)	921 (76.0)	492 (77.8)	515 (78.7)
Cost as limiting factor for regular SMBG, n (%)††	\	\	383 (48.9)	130 (52.4)	438 (49.8)	304 (64.3)	311 (62.6)

n (%) data are presented unless otherwise stated. Percentages were calculated for patients with available data; these varied by each category/wave.

OGLD, oral glucose-lowering drug; SD, standard deviation; SMBG, self-monitoring blood glucose

††Data not available for Wave 2; ††Data not available for Waves 1 and 2

ESM Table 7. Diabetes education (overall population)

Characteristic	*Wave 1 (2005) N=9918*	*Wave 2 (2006) N=17,232*	*Wave 3 (2008) N=12,210*	*Wave 4 (2010) N=5343*	*Wave 5 (2011–12) N=9603*	*Wave 6 (2013–14) N=5479*	*Wave 7 (2016–17) N=6303*
Proportion of patients receiving diabetes education, n (%)	5715 (59.0)	7186 (42.6)	6508 (55.1)	3093 (59.0)	6794 (71.3)	3974 (73.3)	4871 (78.3)
*Person who delivered education, n (%)**							
Peer	0	0	0	160 (5.2)	502 (7.4)	274 (6.9)	326 (6.7)
Nurse	0	0	0	762 (24.6)	1513 (22.3)	839 (21.1)	1348 (27.7)
Certified diabetes educator	0	0	0	467 (15.1)	1042 (15.3)	805 (20.3)	1394 (28.7)
Dietitian/nutritionist	0	0	0	824 (26.6)	1512 (22.3)	660 (16.6)	1385 (28.5)
Physician	0	0	0	2313 (74.8)	5276 (77.7)	3053 (76.8)	3468 (71.3)
*Format of programme, n (%)***							
Structured courses	0	0	0	0	605 (8.9)	415 (10.4)	755 (15.5)
Individual	4361 (77.4)	5484 (78.4)	5388 (83.2)	2441 (80.0)	4865 (71.6)	2567 (64.6)	3163 (65.0)
Group	1309 (23.2)	2298 (32.9)	1708 (26.4)	669 (21.9)	1761 (25.9)	1174 (29.5)	1515 (31.1)

n (%) data are presented unless otherwise stated. Percentages were calculated for patients with available data; these varied by each category/wave.

*Patient could receive education from more than one source; **Patient could undertake education in more than one format

ESM Figure 1. **Insulin therapies by therapy type subgroup**

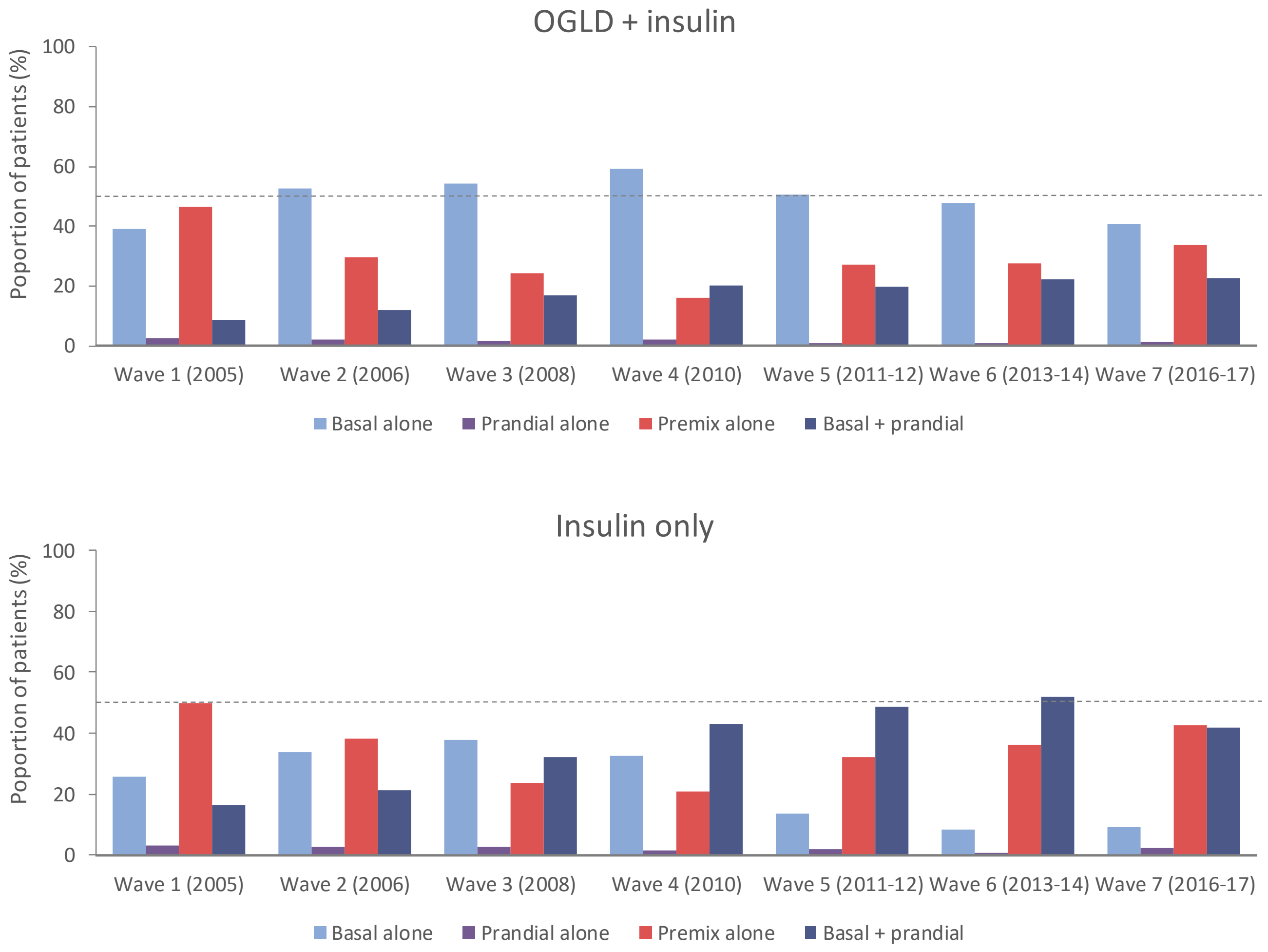

n (%) data are presented unless otherwise stated. Percentages were calculated for patients with available data; these varied by each category/wave.
OGLD, oral glucose-lowering drug
